# Associations Between Race and Survival Outcomes Among Veterans With Head and Neck Cancer in a Racially Diverse Setting

**DOI:** 10.1002/oto2.150

**Published:** 2024-06-11

**Authors:** Amanda R. Walsh, Jonathan P. Giurintano, Jessica H. Maxwell, Anuja H. Shah, Thomas L. Haupt, Andrew E. Wadley, Sandeep R. Kowkuntla, Andy M. Habib, Veranca Shah

**Affiliations:** ^1^ Department of Otolaryngology–Head and Neck Surgery MedStar Georgetown University Hospital Washington District of Columbia USA; ^2^ Department of Otolaryngology–Head and Neck Surgery District of Columbia Veteran's Affairs Medical Center Washington District of Columbia USA; ^3^ Department of Otolaryngology–Head and Neck Surgery University of Pittsburgh Medical Center Pittsburgh Pennsylvania USA; ^4^ Department of Otolaryngology‐Head and Neck Surgery Georgetown University School of Medicine Washington District of Columbia USA; ^5^ Howard University College of Medicine Washington District of Columbia USA

**Keywords:** head and neck cancer, race, survival

## Abstract

**Objective:**

There is limited data on the impact of clinical‐demographic factors on survival outcomes among veterans with head and neck squamous cell carcinoma (HNSCC). This study was undertaken to evaluate the impact of race and other factors on overall survival (OS) in a population of veterans with HNSCC treated with curative intent.

**Methods:**

Demographic and clinical data were collected on veterans with HNSCC treated with curative intent at our institution between 1999 and 2021. The primary outcome was 3‐year OS. Secondary outcomes included treatment delay intervals, including time to treatment initiation (TTI), total package time, and duration of chemoradiation (DCRT).

**Results:**

Of 260 veterans with HNSCC, black veterans had significantly lower 3‐year OS (49.4%) compared to white veterans (65%, *P* = .019). Black veterans were also more likely to experience delays in treatment initiation (median TTI 46 vs 41 days; *P* = .047). Black patients were more likely to receive radiation alone (25.8% [black] vs 8.4% [white]; *P* < .001) and less likely to receive adjuvant therapy if treated surgically (11.1% [black] vs 22.4% [white]; *P* = .004), despite any statistically significant difference in stage of their tumor at presentation (Stage I: 21.2% [black] vs 19.6% [white]; *P* = .372); (Stage IV: 44.4% [black] vs 48.6% [white]; *P* = .487). Other factors associated with worse 3‐year OS included older age (*P* = .023), lower body mass index (*P* = .026), neurocognitive disorder/dementia (*P* = .037), mental health disorders (*P* = .020), hypopharyngeal primary (*P* = .001), higher stage disease (*P* = .002), treatment type (*P* = .001), need for prophylactic gastrostomy tube (*P* = .048) or tracheotomy (*P* = .005), recurrent disease (*P* = .036), persistent disease (*P* < .001), distant metastases (*P* = .002), longer TTI (*P* = .0362), and longer DCRT (*P* = .004).

**Discussion:**

Black race appears to be an independent predictor of 3‐year OS in veterans with HNSCC. Further studies are warranted to determine the factors responsible for disparities in survival.

**Implications for Practice:**

This study evaluated the ways in which race affects survival for US veterans with head and neck cancer. The authors found that black veterans had an increased risk of death compared to white patients, and also experienced delays when receiving treatment.

**Level of Evidence:**

Level IV.

Head and neck squamous cell carcinoma (HNSCC) has the seventh‐highest incidence among malignancies worldwide.^1^ In the United States, the incidence of HNSCC is approximately 3%. Among US veterans, the incidence of HNSCC is approximately 6%, twice as high as the general population.^2^ Multiple clinical and demographic factors including age, sex, race, site, stage at diagnosis, p16 status, smoking, delays in time to treatment initiation (TTI) and total package time (TPT) have been found to impact overall survival (OS) in the civilian population.^3^, ^4^, ^5^, ^6^, ^7^, ^8^, ^9^, ^10^

Black patients have previously been shown to be susceptible to disparities in oncologic care, resulting in diminished survival outcomes. Separate studies have previously reported increased mortality rates and decreased long‐term survival in black patients with advanced oral cavity and oropharyngeal cancers compared to their white counterparts.^11^, ^12^, ^13^, ^14^, ^15^ While studies have demonstrated worse overall and disease‐specific survival in black patients with HNSCC in the civilian population, few studies have evaluated clinical‐demographic factors associated with OS in US veterans treated for HNSCC. Sandulache et al examined the impact of race/ethnicity on veterans diagnosed with laryngeal cancer, concluding that there was high compliance with the standard of care and excellent oncologic outcomes at 3 years irrespective of race.^16^ However, this study was performed at a single VA Medical Center with black patients constituting only 33% of veterans with laryngeal cancer.

Therefore, given the increased incidence of HNSCC in US veterans compared to the civilian population, we sought to determine the impact of race on outcomes among a racially diverse veteran population in Washington DC.

## Materials and Methods

After obtaining approval from our Institutional Review Board, we retrospectively identified veteran patients with HNSCC through the DC Veteran's Affairs Medical Center (VAMC) Department of Otolaryngology's tumor registry. For the purposes of this study, a veteran patient is defined as any individual who received care at the Washington DC VAMC. Veterans diagnosed with HNSCC who were treated with curative intent were included in the study. Patients were excluded if they received all or a portion of their treatment outside the Washington DC VAMC. Other exclusion criteria included a prior history of head and neck cancer and treatment with palliative, noncurative intent due to either the extent of the disease or patient preference.

Demographic data was collected on age at diagnosis, sex, race, body mass index (BMI) at diagnosis, tobacco and alcohol use, housing problems, and comorbidities including neurocognitive disorders, mental health disorders, substance abuse, and chronic pain. Clinical‐pathologic data included tumor subsite (oral cavity, oropharynx, larynx, hypopharynx), tumor stage, date of diagnosis (defined as the date of histopathological confirmation of malignancy from biopsy specimens), treatment types and dates, and occurrence of local‐regional recurrence or distant metastases. The presence and date of supportive surgical procedures, including gastrostomy tube placement, tracheostomy, and dental extraction, were also recorded. Time to last follow‐up and date of death were recorded.

The primary endpoint was 3‐year OS as determined by the date of death from any cause. Patients who were alive with disease or alive with no evidence of disease were only included in this survival analysis if the most recent date of follow‐up was at least 36 months after the date of diagnosis.

The secondary endpoint was to assess 3 quantifiable measurements of treatment delay. First, we determined TTI, defined as the time from the date of diagnosis to the date of surgery or chemoradiation. For patients treated surgically, we also determined TPT, defined as the time from the date of surgery to the completion of adjuvant therapy. TPT is an important consideration as delays in postoperative radiation, defined as the time from date of surgery to the initiation of adjuvant therapy, has a well‐established association with poorer outcomes in patients with HNSCC.^10^ For patients treated with definitive chemoradiation, the duration of chemoradiation (DCRT), defined as the start date of treatment to the completion date of treatment, was also determined. Median TTIs, TPTs, and DCRTs with interquartile ranges (IQRs) were calculated.

Summary statistics, including means, medians, standard deviations, IQR, and proportions (if categorical) on all characteristics, were obtained for the overall sample. *χ* ^2^ and Fisher's exact tests were utilized to analyze the association of categorical variables, while an independent sample *t* test was used to determine the statistical difference among the parametric continuous variables. The Kruskal‐Wallis test was utilized to compare the nonparametric data of 2 or more independent groups.

Kaplan‐Meier estimator curves were used to visualize OS between the 2 groups. In addition, the Log‐rank test for equality of survival functions was used to compare OS between the 2 groups. A multivariable Cox proportional‐hazards model was utilized to test the association between the survival time of subjects and 1 or more of the study variables such as race, radiation versus surgical treatment, stage, primary site, disease persistence, and age. A 2‐sided *P* value less than .05 was considered statistically significant. Statistical Analysis System software version 9.4 (SAS Institute Inc.) was used to perform the analysis.

## Results

Three hundred and twenty veterans with HNSCC were identified who met the inclusion criteria for demographic analysis. Sites of disease were as follows: oropharynx (41.6%), larynx (30.9%), oral cavity (20.3%), hypopharynx (6.3%), and unknown primary (0.9%). The majority of patients had advanced disease at the time of diagnosis, with 46.9% having stage IV disease. 41.9% of patients were treated with surgery ± adjuvant radiation or chemoradiation, while the remaining 58.1% were treated primarily with nonsurgical therapy. The follow‐up period ranged from 18 days to 19.9 years. The vast majority of patients were male (98.6%), and the majority of veterans were black (61.9%). The distribution of comorbidities along with clinical and demographic factors are depicted in Table 1.

**Table 1:** Clinical‐Demographic Characteristics of Study Participants

Characteristic	N (%)
Gender	
Male	316 (98.8)
Female	4 (1.3)
Race	
White	107 (33.4)
Black	198 (61.9)
Asian	1 (0.3)
Other	5 (1.6)
Unanswered	9 (2.8)
Age, years	
<40	1 (0.3)
40‐49	15 (4.6)
50‐59	100 (31.2)
60‐69	123 (38.4)
70‐79	65 (20.3)
>80	15 (4.6)
BMI at diagnosis	
<18.5 (underweight)	45 (14.0)
18.5‐24.9 (healthy weight)	133 (41.5)
25‐29.9 (overweight)	79 (24.6)
30‐34.9 (obesity class I)	33 (10.3)
35‐39.9	19 (5.9)
>40	6 (1.8)
Select comorbidities	
Dementia or neurocognitive disorder	15 (4.7)
Substance abuse disorder	30 (9.4)
Chronic pain	43(13.4)
Mental health disorder	110 (34.4)
Housing problems	23 (7.2)
Alcohol use	271 (84.7)
Tobacco use	293 (91.6)
Primary site	
Oral cavity	65 (20.3)
Oropharynx	133 (41.6)
Larynx	99 (30.9)
Hypopharynx	20 (6.3)
Unknown primary	3 (0.9)
Stage	
1	66 (20.6)
2	42 (13.1)
3	62 (19.4)
4	150 (46.9)
Treatment type	
Surgery	58 (18.1)
Surgery + radiotherapy	30 (9.4)
Surgery + chemoradiation therapy	46 (14.4)
Radiotherapy	65 (20.3)
Chemoradiation therapy	121 (37.8)
Additional performed procedures	
Dental extraction	145 (45.3)
Gastrostomy tube placement	134 (41.8)
Prophylactic tracheostomy	56 (17.5)
Disease status	
Recurrence	53 (16.6)
Persistence	48 (15)
Distant metastasis	44 (13.8)

Sums and percentages of total participants are noted for each variable.

Abbreviation: BMI, body mass index.

To assess 3‐year OS, we identified 260 patients which included those who were alive at 36 months after initial diagnosis (n = 141) and those who died prior to 36 months (n = 119). Patients who were alive at their last follow‐up but had less than 36 months of follow‐up were excluded from survival analysis (n = 60). Of the 320 veterans with HNSCC, there were no patients identified to be lost to follow‐up within the 36‐month period. 3‐year OS for the entire cohort was 54.2%. Factors significantly associated with death within 3 years of treatment included older age at the time of diagnosis (65.3 vs 62.6 years; *P* = .023) and lower BMI at diagnosis (23.6 vs 25.2; *P* = .026). Several other factors were found to have a statistically significant association with worse OS at 3 years, as seen in Table 2.

**Table 2:** 3‐Year OS for Veterans With HNSCC

	Alive, n = 141	Dead, n = 119	
Characteristic	N (%)	N (%)	*P* value
Gender			.109
Male	138 (53.7)	119 (46.3)	
Female	3 (100)	0 (0)	
Race			**.019**
White	52 (65.0)	28 (35.0)	
Black	89 (49.4)	91 (50.6)	
Age, year			
Average (SD)	62.6 ± 9.2	65.3 ± 9.2	**.023**
BMI at diagnosis			
Average (SD)	25.2 ± 5.6	23.6 ± 5.6	**.026**
<18.5 (underweight)	16 (39.0)	25 (61.0)	**.033**
18.5‐24.9 (healthy weight)	57 (50.4)	56 (49.6)	.282
25‐29.9 (overweight)	39 (66.1)	20 (33.9)	**.037**
30‐34.9 (obesity class I)	17 (63.0)	10 (37.0)	.336
35‐39.9 (obesity class II)	10 (71.4)	4 (28.6)	.184
40 (obesity class III/morbid obesity)	0 (0)	2 (100)	.120
Select comorbidities			
Dementia or neurocognitive disorder	4 (30.8)	9 (69.2)	**.037**
Substance abuse disorder	13 (52.0)	12 (48.0)	.814
Chronic pain	19 (65.5)	10 (34.5)	.196
Mental health disorder	42 (44.7)	52 (55.3)	**.020**
Housing problems	7 (38.9)	11 (61.1)	.176
Alcohol use	120 (52.6)	108 (47.4)	.302
Tobacco use	127 (53.1)	112 (46.9)	.233
Primary site			**.003**
Oral cavity	30 (54.5)	25 (45.5)	.950
Oropharynx	54 (51.4)	51 (48.6)	.455
Larynx	52 (65.0)	28 (35.0)	**.020**
Hypopharynx	3 (16.7)	15 (83.3)	**.001**
Unknown primary	2 (100.0)	0 (0)	.192
Stage			**.002**
1	35 (77.8)	10 (22.2)	**.001**
2	24 (64.9)	13 (35.1)	.161
3	27 (55.1)	22 (44.9)	.892
4	55(42.6)	74 (57.4)	**<.001**
Treatment type			**.001**
Surgery alone	35 (77.8)	10 (22.2)	**.001**
Surgery + adjuvant radiation	16 (66.7)	8 (33.3)	.199
Surgery + adjuvant chemoradiation	22 (59.5)	15 (40.5)	.491
Radiation alone	23 (45.1)	28 (54.9)	.144
Chemoradiation alone	45 (43.7)	58 (56.3)	**.006**
TTI, days			
Median (IQR) TTI for all patients	41 (23‐65)	48 (29‐81)	**.038**
TPT, days			
Median (IQR) TPT for patients treated surgically	100.5 (88‐115)	106 (91‐135)	.197
DCRT, days			
Median (IQR) for patients treated with definitive DCRT	52 (45.5‐57)	59 (49‐68)	**.004**
Additional performed procedures			
Dental extraction	60 (49.6)	61 (50.4)	.161
Gastrostomy tube placement	46 (46.5)	53 (53.5)	**.048**
Prophylactic tracheostomy	16 (35.6)	29 (64.4)	**.005**
Disease status			
Recurrence	19 (40.4)	28 (59.6)	**.036**
Persistence	7 (17.1)	34 (82.9)	**<.001**
Distant metastasis	12 (28.6)	30 (71.4)	**.002**

Sums and percentages of participants who fall into each category are noted. *χ* ^2^ and Fischer's exact tests were used to analyze the association of categorical variables, while independent sample *t* tests were used to determine the statistical difference among parametric continuous variables. For nonparametric data (TTI, TPT, and DCRT), the Kruskal‐Wallis test was used to compare medians with IQRs. A *P* < .05 was considered significant and all significant factors are noted in bold text

Abbreviations: DCRT, duration of chemoradiation; HNSCC, head and neck squamous cell carcinoma; IQR, interquartile range; OS, overall survival; TPT, total package time; TTI, time to initiation.

**Figure 1:**
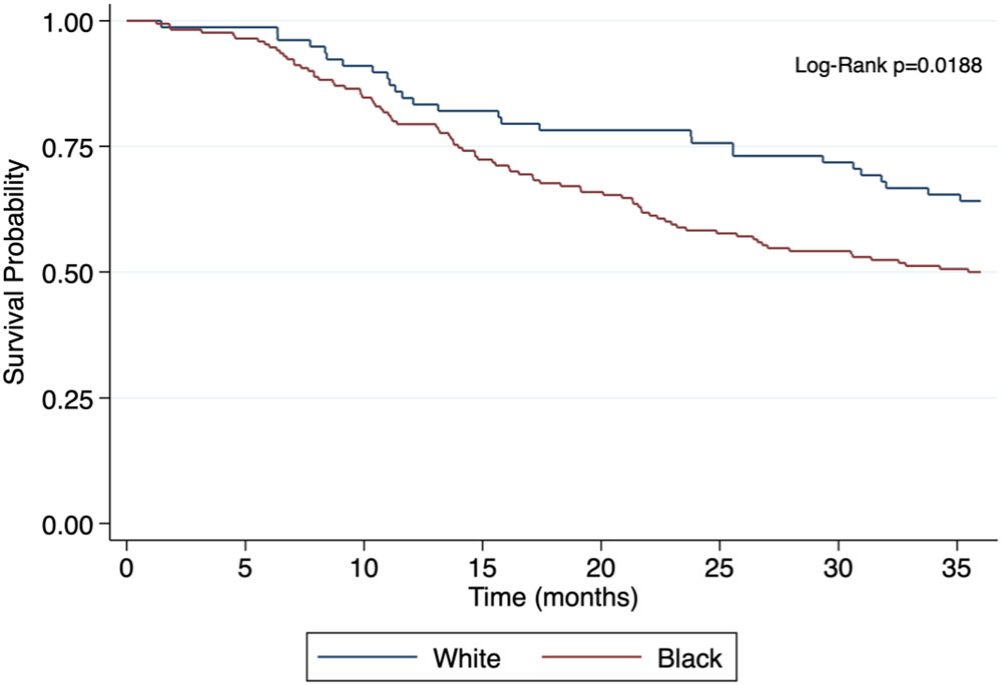
Kaplan‐Meier curve for 3‐year overall survival in black and white veterans with head and neck squamous cell carcinoma. Three‐year overall survival is significantly worse in black veterans (*P* = .0188).

Black veterans had worse 3‐year OS compared to white veterans (49.4% vs 65%; *P* = .019). A Kaplan‐Meier curve for 3‐year OS based on race was constructed (see Figure 1). Multivariable analysis with Cox proportional hazard ratio (HR) adjusted for covariables such as race, radiation vs surgical treatment, stage, disease persistence, and age was performed to estimate the risk of overall mortality; this demonstrated that race remained a significant prognostic factor, with black veterans having a higher HR for mortality compared to whites (HR = 1.712 [95% confidence interval, CI : 1.038‐2.872]; *P* = .035) (Table 3). Given the confirmation that black veterans had worse 3‐year OS, additional investigation was performed to evaluate the association between race and other clinical demographic factors in this cohort (Table 4).

**Table 3:** Factors Associated With Statistically Significant Increased Hazard of Death in Veterans With HNSCC

Characteristic	HR	95% CI	*P* Value
Black race	1.727	1.038‐2.872	.035
Radiation alone	2.510	1.240‐5.040	.010
Stage 3 disease	2.657	1.415‐4.989	.002
Persistent disease	4.54	2.530‐8.160	<.001
Age (per year)	1.032	1.008‐1.060	.009

A multivariable Cox proportional‐hazards model was utilized to test the association between the clinical factors and survival time of subjects. Factors with significant (*P* < .05) HRs for death are listed.

Abbreviations: CI, confidence interval; HNSCC, head and neck squamous cell carcinoma; HR, hazard ratio.

**Table 4:** Clinical‐Demographic Factors of Veterans With HNSCC by Stratified by Race

	Black, n = 198	White, n = 107	
Characteristic	N (%)	N (%)	*P* value
Gender			.301
Male	194 (98)	107 (100)	
Female	4 (2)	0 (0)	
Age, year			
Average (SD)	63.8 ± 9.0	64.1 ± 9.2	.789
BMI at diagnosis			
Average (SD)	23.9 ± 5.6	26.8 ± 6.2	**<.001**
<18.5 (underweight)	35 (17.7)	9 (8.4)	**.014**
18.5‐24.9 (healthy weight)	87 (43.9)	39 (36.4)	.205
25‐29.9 (overweight)	48 (24.2)	28 (26.2)	.645
30‐34.9 (obesity class I)	17 (8.6)	14 (13.1)	.215
35‐39.9 (obesity class II)	9 (4.5)	9 (8.4)	.086
40 (obesity class III/morbid obesity)	1 (0.5)	4 (3.7)	**.034**
Select comorbidities			
Dementia or neurocognitive disorder	12 (6.1)	3 (2.8)	.105
Substance abuse disorder	27 (13.6)	3 (2.8%)	**.001**
Chronic pain	29 (14.6)	14 (13.1)	.708
Mental health disorder	69 (34.8)	37 (34.6%)	.962
Housing problems	18 (9.0)	5 (4.6%)	.082
Alcohol use	178 (89.9)	82 (76.6)	**.001**
Tobacco use	185 (93.4)	95 (88.8)	.079
Primary site			**.002**
Oral cavity	34 (17.2)	29 (27.1)	**.021**
Oropharynx	76 (38.4)	49 (45.8)	.105
Larynx	72 (36.4)	22 (20.6)	**.002**
Hypopharynx	16 (8.1)	4 (3.7)	.072
Unknown primary	0 (0)	3 (2.8)	**.018**
Stage			.737
1	42 (21.2)	21 (19.6)	.372
2	31 (15.7)	11 (10.3)	.097
3	36 (18.2)	23 (21.5)	.485
4	88 (44.4)	52 (48.6)	.487
Treatment type			**.002**
Surgery alone	35 (17.7)	22 (20.6)	.269
Surgery + adjuvant radiation	17 (8.6)	12 (11.2)	.228
Surgery + adjuvant chemoradiation	22 (11.1)	24 (22.4)	**.004**
Radiation alone	51 (25.8)	9 (8.4)	**<.001**
Chemoradiation alone	73 (36.9)	40 (37.4)	.929
TTI, days			
Median (IQR) TTI for all patients	46 (27‐74)	40 (22‐63)	**.047**
TPT, days			
Median (IQR) TPT for patients treated surgically	104 (91‐123)	105.5 (88‐124)	.991
DCRT, days			
Median (IQR) for patients treated with definitive DCRT	56 (44‐63)	56 (50‐63)	.433
Additional performed procedures			
Dental extraction	107 (54)	35 (32.7)	**<.001**
Gastrostomy tube placement	79 (39.9)	48 (44.9)	.201
Prophylactic tracheostomy	42 (21.2)	15 (14)	.062
Disease status			
Recurrence	36 (18.2)	14 (13.1)	.126
Persistence	27 (13.6)	18 (16.8)	.227
Distant metastasis	33 (16.7)	12 (11.2)	.100

Sums and percentages of participants who fall into each category are noted. *χ* ^2^ and Fischer's exact tests were used to analyze the association of categorical variables, while independent sample *t* tests were used to determine the statistical difference among parametric continuous variables. For nonparametric data (TTI, TPT, and DCRT), the Kruskal‐Wallis test was used to compare medians with IQRs. A *P* < .05 was considered significant and all significant factors are noted in bold text.

Abbreviations: BMI, body mass index; DCRT, duration of chemoradiation; HNSCC, head and neck squamous cell carcinoma; IQR, interquartile range; TPT, total package time; TTI, time to initiation.

Black veterans were more likely to have lower BMI than white veterans (average [SD] = 23.9 ± 5.6 vs 26.8 ± 6.2; *P* < .001). Black veterans were more likely to suffer from substance abuse disorders (13.6% vs 2.8%; *P* = .001) and to report alcohol use compared to white veterans (89.9% vs 76.6%; *P* = .001). There was no statistically significant difference in the proportion of patients who reported tobacco use when stratified by race (93.4% vs 88.8%; *P* = .079). Of note, there was no statistically significant difference in the clinical stage at presentation (Table 4).

As patients treated with surgery alone had significantly better 3‐year OS compared to patients treated with chemoradiation, we sought to determine whether black patients received different types of treatment compared to their white counterparts. Black veterans were significantly more likely to receive radiation alone (25.8% vs 8.4%; *P* < .001) and significantly less likely to receive triple modality therapy, surgery plus chemoradiation therapy (11.1% vs 22.4%) (Table 4). Interestingly, this is in spite of the fact that black and white patients had no statistically significant difference in stage of their tumor at presentation (Stage I: 21.2% [black] vs 19.6% [white];  = .372); (Stage IV: 44.4% [black] vs 48.6% [white]; *P* = .487; Table 4). A multivariable analysis showed that the HR for death for patients treated with radiation alone was 2.510 (95% CI: 1.249‐5.042; *P* = .010) (Table 3). Comparing primary sites, white veterans had significantly increased rates of oral cavity cancer compared to black veterans (27.1% vs 17.2%), whereas black veterans had significantly increased rates of laryngeal cancer compared to white veterans (36.4% vs 20.6%).

**Figure 2:**
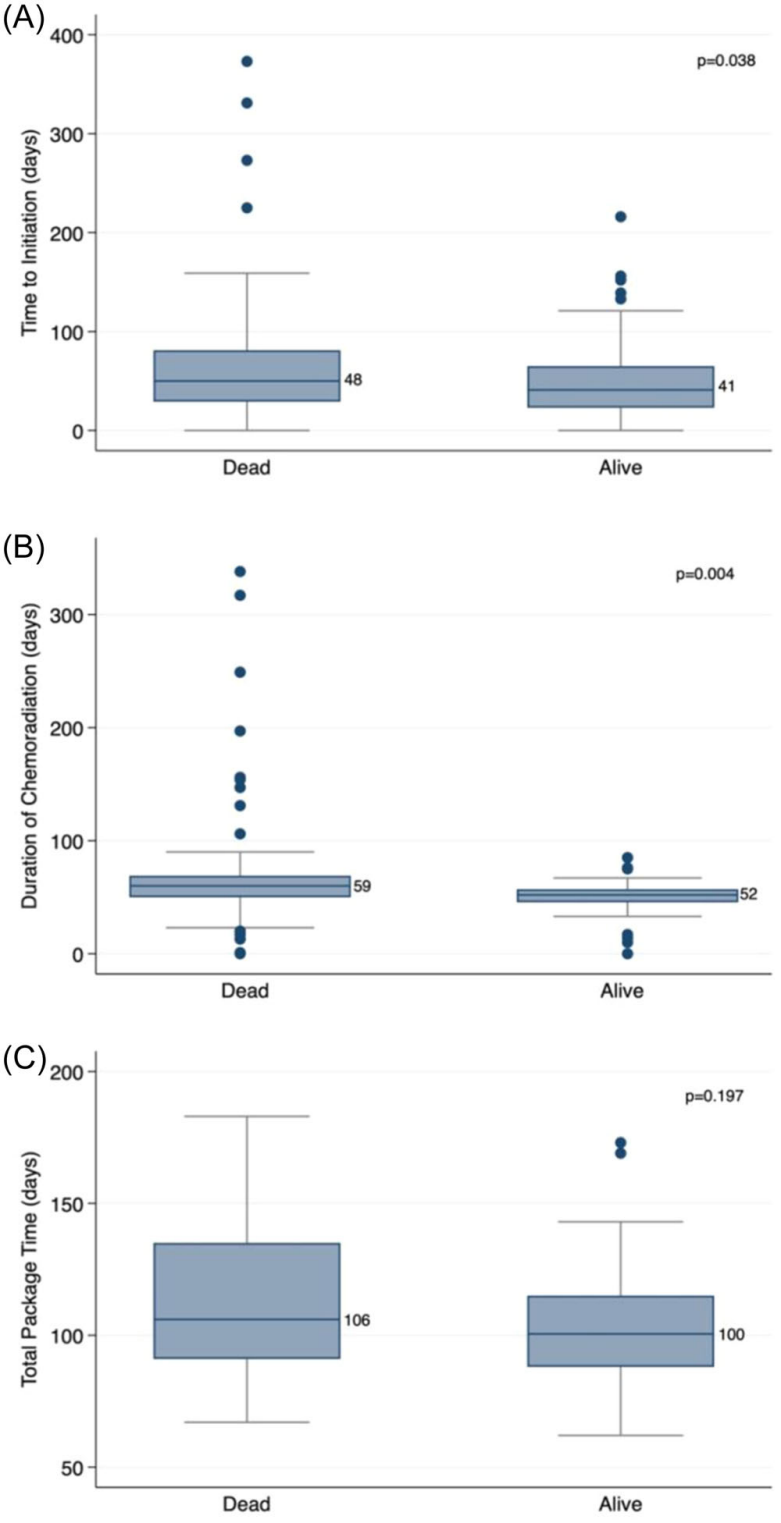
Impact of treatment delay on 3‐year overall survival in veterans with head and neck squamous cell carcinoma. (A) Time to initiation, (B) duration of chemoradiation, and (C) Total package time. Median time to initiation with interquartile ranges are shown for the 3 treatment intervals; *P* values are displayed. Longer time to initiation and duration of chemoradiation are associated with worse survival in veterans with head and neck squamous cell carcinoma.

The secondary endpoint for this study was assessing variables measuring treatment delay, including TTI, TPT, and DCRT. Our results showed that delays in treatment affected OS; longer TTIs were associated with worse survival, with the median (IQR) TTI being 41 (23‐65) days for patients alive at 3 years and 48 (29‐81) days for patients who died before this time point (*P* = .038). Similarly, longer DCRTs were associated with worse survival, with the median (IQR) DCRT being 52 (45.5‐57) days for patients alive at 3 years and 59 (49‐68) days for patients who died before this time point (*P* = .004). TPT did not have a statistically significant association with OS in this cohort (*P* = .197) (Figure 2, Table 2).

With regard to race, the median TTI for black patients was 6 days longer than for white patients (46 vs 40 days; *P* = .047). Black veterans were more likely to require dental extraction than white veterans (54% vs 32.7%; *P* < .0001), a factor that may delay TTI for patients requiring radiation. The median TPT for patients treated surgically was not significantly different when comparing black versus white patients. Similarly, there was not a statistically significance difference in median DCRT for patients treated with DCRT when stratifying patients by race (Table 4).

## Discussion

At the Washington DC VAMC, black veterans represent 61% of patients diagnosed with HNSCC despite only representing 12% of the total US veteran population. Whereas previous studies such as Sandulache et al showed no significant difference in 3‐year OS between black and white veterans diagnosed with laryngeal cancer, in our institution, black veterans were found to have worse 3‐year OS compared to white veterans.^16^ Despite there being no significant differences in disease stage at time of diagnosis in blacks compared to whites, black veterans had a significantly longer time to initiation of treatment compared to white veterans, a potentially addressable treatment disparity that was associated with decreased survival.

This disparity in TTI and OS is most likely multifactorial. Our findings demonstrated that black patients were more likely to have comorbidities associated with decreased survival outcomes, such as lower BMI and alcohol and substance abuse. While beyond the scope of this study, differences in socioeconomic status, access to care, patient beliefs and preferences, and potential biases may also have contributed to black veterans' worse survival outcomes in our study. The higher percentage of black patients in our cohort compared to prior studies may have allowed for this disparity to become statistically evident where it was not previously recognized in other VAMCs as demonstrated by Sandulache et al.^16^

In our cohort, black patients were more likely to receive radiation alone, whereas whites were more likely to receive triple modality therapy. These discrepancies may be explained by the significantly higher incidence of laryngeal cancer in black patients and the significantly higher incidence of oral cavity cancer in white patients, as patients with early stage laryngeal cancers are more likely to undergo treatment with single‐modality radiation compared to other tumor sites, while surgical management represents the standard of care for oral cavity cancers. Deference of surgical treatment to nonsurgical management may stem from patient beliefs and aversion to surgical treatment or life‐altering surgical treatment, particularly, in the case of laryngectomy for advanced laryngeal cancer. Another study has shown that black patients with T4 laryngeal cancer, a disease for which treatment consisting of total laryngectomy followed by radiation/chemoradiation is the standard of care, were significantly more likely to select primary radiotherapy as opposed to total laryngectomy based on a series of hypothetical scenarios encompassing a range of 3‐year survival and speech utility when compared to their white counterparts.^17^ This preference for nonsurgical treatment has also been shown outside of the head and neck. A prospective study of black and white veterans with carotid stenosis previously showed that black veterans expressed a higher aversion to undergoing carotid endarterectomy and were less likely to pursue surgical treatment.^18^ In our cohort, the increased incidence of laryngeal cancer in black veterans with the increased propensity to undergo treatment consisting of radiation alone, possibly in the setting of advanced T3 or T4 disease, may potentially explain observed differences in 3‐year OS.

Studies have shown that delays in treatment can result in pathologic upstaging, increased mortality, and increased likelihood of recurrence, however, this finding has not been elucidated in a veteran population.^19^, ^20^, ^21^ Our work demonstrates that prolonged TTI and DCRT are associated with decreased OS in veterans with HNSCC, thus reinforcing that timely initiation and completion of treatment are critical to delivering quality care. This delay in treatment initiation may be explained in part by an increased likelihood of requiring dental extraction prior to radiation. Since black veterans were more likely to present with laryngeal cancer compared to white veterans, they would more often require dental extraction prior to treatment. On the contrary, oral cavity cancers which were more often seen in white veterans, may obtain dental extractions during the initial surgical treatment, therefore avoiding potential dental extraction delays.

There are several limitations of this study that are important to note. First, this is a retrospective study of veterans with HNSCC over 2 decades, therefore susceptible to selection bias and errors in charting and extracting data. Given the timespan of this study, there may have been changes in treatment recommendations, patient and physician preferences, and access to care over time. The most robust interval offered by the dataset was 36 months to assess 3‐year OS, limiting the study's ability to assess 5‐year OS. There are also a number of factors that are difficult to quantify and measure in this study, such as socioeconomic status and financial toxicity. These are important aspects of head and neck cancer care and outcomes, potentially confounding our results. Ultimately, this study is unique in that it represents the impact of race on outcomes in a highly diverse veteran population with nearly two‐thirds of the patients with HNSCC identifying as black. Further studies are necessary to examine on a more granular level the racial and health disparities among veterans with HNSCC.

## Conclusions

Our study demonstrated that race was an independent predictor of OS in veterans with HNSCC at a single, racially diverse VAMC. Black veterans were more likely to be diagnosed with laryngeal cancer, more likely to be treated with radiotherapy alone, and less likely to receive triple modality therapy than white patients. In addition, black patients experienced delays in treatment initiation when compared to white patients, potentially representing an addressable health disparity in the veteran population. Further studies are warranted to determine the factors responsible for such disparities and potential interventions to ultimately improve outcomes among veterans with HNSCC.

## Author Contributions

**Amanda R. Walsh**, study design, table/figure creation, manuscript writing; **Jonathan P. Giurintano**, study design, manuscript review; **Jessica H. Maxwell**, study design, manuscript writing; **Anuja H. Shah**, manuscript writing, manuscript review, manuscript submission; **Thomas L. Haupt**, data collection, data analysis, manuscript writing; **Andrew E. Wadley**, data collection, data analysis, manuscript writing; **Sandeep R. Kowkuntla**, data collection, manuscript writing; **Andy M. Habib**, study design, data collection; **Veranca Shah**, data collection.

## Disclosures

### Competing interests

None.

### Funding source

None.
